# Dental Collegiate Education

**Published:** 1869-11

**Authors:** 


### EDITORIAL DEPARTMENT.

*Dental Collegiate Education.*—The Dental Colleges are again in operation, the Annual Sessions having commenced with infirmary practice and preliminary lectures, about the middle of last month, and the regular lectures on the first day of the present month.

We learn that at most of these institutions the prospects for an increased number of students over last year are very encouraging; and in some of them the standard of qualification has been raised, and every available effort being made to render the degree they confer a true honor and mark of fitness, and not one of disgrace.

Those who are, or have been, engaged in teaching will understand the responsibilities and labor, physical as well as mental, which is attached to a professorship. The dental teacher must search for error as well as for truth, in order that he may tread down the one and embrace the other. All kinds of systems must come under his observation, and nothing be allowed to escape his attention, in order that his understanding may be enlarged and his ability to confer knowledge be increased.

That our Dental Colleges, like the Medical, are not all they should be is an indisputable fact, but the time has come for a liberal reform. There may have been and no doubt was a time when the practical difficulties in the way rendered it impossible to adopt a high and rational system of instruction, and to exact more than a miserably low standard of qualification. But such a time has passed away, and important changes for the better have been adopted in regard to the course and range of study, and the standard of qualification. It is not expected that a student, on receiving the degree of D.D.S. should be an accomplished dentist, perfect in all that pertains to the practice of his profession, but it is expected that when he enters into regular practice, a broad line will exist between himself and the quack. For the benefit of those of our readers who are uninformed in regard to what is taught in Dental Colleges, we give the following summary of the course pursued in the institution with which we are connected:

The lectures on Pathology and Therapeutics teach the principles of general pathology, also the special pathology, diagnosis, and treatment of those diseases which involve the structures of the mouth and adjacent parts, and the local effect upon these organs of general, constitutional and hereditary disease. All the prominent articles of the materia medica are accurately described, illustrated by specimens and botanical plates, and accompanied by their appropriate therapeutical indications.

The lectures on Dental Science and Mechanism teach the true bearing of scientific culture upon Dental art; its absolute necessity, and the relative importance of the several sciences—the connection between dentistry and general medicine and surgery —and the mechanical, artistic and æsthetic elements of dentistry. which distinguish it from all other branches of the *Ars medendi.* All details of dental mechanism are explained and demonstrated. The tendency to reject older methods for untried novelties guarded against, whilst all discoveries and improvements, real or supposed are described. The lecturer's aim is to make plain both the method and the rule; but, more than this, to give so clearly the scientific principles thereof, that the student shall be master to the rule and not the slave to a set of formulas.

The lectures on the Practice of Dental Surgery, comprise all the practical details connected with the etiology, pathology and treatment of the morbid conditions and structural changes of the teeth, gums, alveolar processes, and maxillary sinus; the nature, prevention and treatment of salivary accretions, of exposed and diseased nervous pulp; extraction of the teeth, and the use of anæsthetic agents for dental operations; materials used in filling teeth, such as plain and adhesive gold foil, crystal, shred and plastic gold, &c.,and the various methods of introducing the same: form, manner of using and the art of tempering instruments; correction of irregularities of the dental arch; dental hygiene, &c. All the principles and rules laid down are put in practice by students in the Infirmary, under the direction of the Professor of this chair, and the constant daily supervision of the Demonstrator of Operative Dentistry.

It is the aim of the Professor of Chemistry to give an accurate general idea of the principles of the science, dwelling especially upon those points which are of peculiar interest to the dental student. The physiological relations of chemistry are unfolded as far as practicable, leaving particular details to the Professor of Physiology. Careful attention is paid to the chemistry of the metals, and of the porcelain materials used in Dentistry; also to the vital chemistry of anæsthetics. A subject so extensive as organic chemistry, can of course be only partially considered.

But the special chemistry of the mouth, the stomach and the intestinal tube are fully treated. The course is as far as practicable, one of applied chemistry.

The science of Anatomy is treated in its application to Dental Surgery. Its subdivisions, microscopical and comparative receive appropriate attention, together with the chief object of the course, the study of Human Regional Anatomy. Thus, a general survey of the subject is taken, sufficient to enable the student to acquire a comprehensive knowledge of the science. The convenient and well arranged Dissecting Rooms in the College Building, afford ample opportunity for the study of Practical Anatomy, of which it is hoped every student will avail himself.

The rapid advance of Dental Surgery, necessitated a division in the chair of Anatomy and Physiology. This necessity was promptly met by the Faculty several years ago, and a distinct chair and professorship assigned to Physiology. The course of lectures embrace, to a certain extent, Anatomy, general, comparative and microscopical, so that the physical character of organs, and the physical principles involved in their action may be fresh in the memory of the student at the time of description of their functions and their physiological relations. The physiology of the Dental organs is very distinctly set forth. Special attention is paid to the nervous system. Digestion, normal and abnormal, is thoroughly investigated, and the importance of oral digestion clearly defined, as being the first step in that process upon the perfection of which the remaining acts mainly depend. Lectures are given on Hygiene and Dietetics. The lectures on Anatomy, Physiology and Chemistry are as thorough as in any medical college, and special attention is also paid to the Microscopical Anatomy of the dental structures.

In the department of practice no pains are spared to make arrangements for the acquirement of practical skill commensurate with the importance of this branch of tuition. From the middle of October until the close of the session, the Infirmary is open every day.

In regard to Practical Anatomy, the Dissecting Rooms in the college building are superior to those in the majority of medical colleges, and contain every facility for a thorough study of the human body.

Throughout the entire session weekly examinations are held by each Professor on the lectures delivered during the week, and a record kept of the proficiency of each student. These records are consulted at the final examinations of the members of the graduating class, and are taken into account in summing up the votes. Reports are also required from each of the Demonstrators who also keep records of all the operations performed in the Infirmary and Laboratory.

